# Verification of immunology-related genetic associations in BPD supports *ABCA3* and five other genes

**DOI:** 10.1038/s41390-021-01689-y

**Published:** 2021-08-31

**Authors:** Felix Blume, Holger Kirsten, Peter Ahnert, Trinad Chakraborty, Arnd Gross, Katrin Horn, Mohammad Reza Toliat, Peter Nürnberg, Eva-Maria Westenfelder, Wolfgang Goepel, Markus Scholz

**Affiliations:** 1 Institute for Medical Informatics, Statistics and Epidemiology (IMISE), associated partner of the German Center for Lung Research (DZL), University of Leipzig, Leipzig, Germany; 2 LIFE - Leipzig Research Centre for Civilisation Diseases, University of Leipzig, Leipzig, Germany; 3 Institute for Clinical Immunology and Transfusion Medicine of University Hospital Giessen, Justus Liebig University of Giessen, Giessen, Germany; 4 Cologne Center for Genomics (CCG), University of Cologne, Cologne, Germany; 5 Center for Cross-age Palliative Medicine and Pediatric Pain Therapy of Saarland University Hospital, Saarland University, Saarland, Germany; 6 Neonatology and Pedriatric ICU of University Hospital of Schleswig-Holstein, University of Kiel and Lübeck, Lübeck, Germany

## Abstract

**Background:**

Inflammatory processes are key drivers of bronchopulmonary dysplasia (BPD), a chronic lung disease in preterm infants. In a large sample, we verify previously reported associations of genetic variants of immunology-related genes with BPD.

**Methods:**

Preterm infants with a gestational age ≤32 weeks from PROGRESS and the German Neonatal Network (GNN) were included. Through a consensus case/control definition, 278 BPD cases and 670 controls were identified. We identified 49 immunity-related genes and 55 single-nucleotide polymorphisms (SNPs) previously associated with BPD through a comprehensive literature survey. Additionally, a quantitative genetic association analysis regarding oxygen supplements, mechanical ventilation, and continuous positive air pressure (CPAP) was performed.

**Results:**

Five candidate SNPs were nominally associated with BPD-related phenotypes with effect directions not conflicting the original studies: rs11265269-*CRP*, rs1427793-*NUAK1*, rs2229569-*SELL*, rs1883617-*VNN2*, and rs4148913-*CHST3*. Four of these genes are involved in cell adhesion. Extending our analysis to all well-imputed SNPs of all candidate genes, the strongest association was rs45538638-*ABCA3* with CPAP (*p* = 4.9 × 10^−7^, FDR = 0.004), an ABC transporter involved in surfactant formation.

**Conclusions:**

Most of the previously reported associations could not be replicated. We found additional support for SNPs in *CRP*, *NUAK1*, *SELL*, *VNN2*, and *ABCA3*. Larger studies and meta-analyses are required to corroborate these findings.

**Impact:**

- Larger cohort for improved statistical power to detect genetic associations with bronchopulmonary dysplasia (BPD).
- Most of the previously reported genetic associations with BPD could not be replicated in this larger study.
- Among investigated immunological relevant candidate genes, additional support was found for variants in genes *CRP*, *NUAK1*, *SELL*, *VNN2*, and *CHST3*, four of them related to cell adhesion.
- rs45538638 is a novel candidate SNP in reported candidate gene ABC-transporter *ABCA3*.
- Results help to prioritize molecular candidate pathomechanisms in follow-up studies.

## Introduction

The life expectancy of preterm infants is continuously increasing due to improved clinical treatment. However, this comes at the cost of an increase in the incidence of bronchopulmonary dysplasia (BPD), especially in preterm infants with low gestational age (GA).^1^ For a GA of 22–25 weeks, incidence of BPD was reported to be up to 80%.^2^ At the current state, BPD is the most common chronic lung disease in preterm infants^3^ and a primary reason for their morbidity and mortality.^4^ Beside low GA, low birth weight (BW), especially if <1500 g, is a well-established risk factor^5^ for the development of BPD. The pathophysiology of the disease is the result of an extremely immature lung and is characterized by subsequent reparative processes resulting in vascular and alveolar underdevelopment^6^ as well as fibrotic remodeling of the lung’s anatomic structures.^7^ These processes can result in reduced gas exchange and hence a reduced lung function. BPD is believed to have a strong genetic background with heritability estimated from twin studies between 50 and 80%.^3^ Hence, six genome-wide association studies (GWASs; none resulting in genome-wide significant associations)^8–13^ as well as several candidate gene studies have been conducted so far (see Supplementary Table 1 for references of included associated candidate single-nucleotide polymorphisms (SNPs)). A major focus of reported candidate studies were genes involved in immune processes resulting from oxidative stress or infections promoted by the general underdeveloped state of the lung.^14^ These processes are believed to play an important role in the development of BPD due to their profound deleterious effects. For example, immune cells, such as macrophages or neutrophils, release oxygen radicals and other inflammatory mediators as a response to infection by bacteria or other exogenous organisms. These molecules can impair pulmonary tissue and further increase pro-inflammatory effects.^1^ Other immune-related genes such as surfactant proteins have multiple effects: On the one hand, they affect alveolization and capillary morphology.^15,16^ On the other, they are associated with immunological processes including accumulation of mast cells in the trachea and antiviral activity of the immune system.^17, 18^ Typically, candidate gene studies published in the past were limited by small sample sizes. Thus, replication of their findings in independent studies is required. Moreover, reported studies were typically limited regarding the number of considered candidate markers per gene. Therefore, our aim was to comprehensively evaluate previously reported immune-related candidate SNPs in a large sample of BPD cases and controls. Going beyond this replication analysis, we analyzed the respective candidate genes in more detail considering additional SNPs of these genes (see Table 1 for an overview of all the included SNPs).

**Table 1:** Overview of candidate SNPs retrieved from previous studies and included in our replication analysis.

Gene	Subgroup	Rs-id	SIGN. LITERATURE (*p* value)^ref.^	NOT SIGN. LITERATURE (*p* value)^ref.^	Power^a^ (*α* = 0.05)
ABCA3	Extracellular processes	rs13332514	(0.002)^52^	—	1
ACE		rs8066114	(5.0 × 10^−3^)^63^	(0.98)^8^	
MMP16		rs2664352	(0.013)^64^	(0.185)^59^	
		rs2664349	(0.047)^64^	—	
VEGF		rs699947	(0.0138)^65^	(0.29)^8^	
		rs833061	(0.013)^66^	(0.3)^8^	
SFTPB		rs2077079	(0.018)^67^		
SFTPD		rs2243639	(0.005)^67^	—	
		rs721917	(0.014)^67^; (0.03)^63^	(0.81)^8^	
NFE2L2		rs6721961	(0.0007)^68^	—	1
NQO1		rs1800566	(0.023)^68^	—	0.7
MBL2	Recognition	rs1800450	(0.001)^69^; (0.0068)^7^	(0.43)^70^	1
		rs11003125	(0.02)^70^	(0.57)^8^	
		rs7096206	(0.0372)^7^	—	1
TIRAP		rs8177374	(0.04)^71^	(0.77)^8^	1
TLR4		rs11536898	(0.01)^72^	—	0.91
TLR5		rs5744168	(0.03)^71^	(0.19)^8^	0.96
TLR6		rs5743827	(0.03)^72^	—	0.97
TLR10		rs11096955	(0.0149)^65^	(0.21)^8^	
PSMF1		rs2235587	(0.00104)^13,b^	—	0.42
CRP		rs11265269	(5.3 × 10^−5^)^12,b^	—	0.97
		rs3093059	(0.0065)^12,b^	—	0.90
CARD17/CASP1P1	Cytokines	rs11226613	(5 × 10^−6^)^8^^,b^	—	0.44
IFG		rs2430561	(0.049)^73^	(0.7)^8^	
IL18		rs360721	(<0.001)^74^	—	1
IL18R1		rs3771171	(6.33 × 10^−6^)^75^	—	1
IL18RAP		rs3771150	(8.31 × 10^−7^)^75^	—	1
MIF		rs755622	(0.04)^76^	(0.98)^8^	1
TNF		rs1799724	(0.0085)^65^	(0.17);^77^ (0.71)^8^	
		rs361525	(0.026)^78^	(0.132)^79^	
FGFR-4	Cellular processes	rs1966265	(0.023)^59^	—	
GSTP-1		rs1695	(0.05)^80^	—	1
IPO13		rs4448553	(0.04)^81^	—	1
NFKBIA		rs2233409	(0.024)^82^	—	
		rs2233406	(0.026)^82^	—	
VDR		rs2228570	(0.004)^83^	—	0.98
		rs731236	(0.04)^83^	—	1
WDR45L		rs8082435	(0.008752)^49^	—	
ERLEC1		rs2542571	(0.00024^)^^13^^,b^	—	0.33
TRIM46		rs3814316	(0.00052)^13^^,b^	—	0.33
PRKRA		rs62176107	(0.00086)^13^^,b^	—	0.44
		rs77419724	(0.00014)^13^^,b^		0.5
BPIFB1		rs1078761	(0.00102)^13^^,b^	—	0.28
CHST3	Cell adhesion	rs4148913	(6.82 × 10^−5^)^8^^,b^	—	0.36
NOS		rs1799983	(0.02)^84^	—	0.99
		rs2070744	(0.04)^84^; (0.05)^85^	—	0.99
SELL		rs2229569	(0.04)^86^	(>0.05)^8^	1
VNN2		rs1883617	(0.00101)^84^^,b^	—	0.25
NUAK1		rs1427793	(1.09 × 10^−6^)^49^	—	
KITLG	Other	rs11104948	(9 × 10^−6^)^87^	—	0.99
		rs4842477	(0.015)^87^	—	0.97
		rs10858753	(0.016)^87^	—	0.91
		rs11104906	(0.017)^87^	—	0.97
		rs17424193	(0.020)^87^	—	0.96
		rs869408	(0.030)^87^	—	0.95

We consider SNPs for which at least one nominally significant association was reported. SIGN. LITERATURE and NOT SIGN. LITERATURE: literature results were stratified by the presence of significant associations with BPD or related phenotypes and the respective reported *p* value is shown. The reference is provided as superscript. The power to replicate significant findings is shown in the last column.

*SIGN.* significant, *ref.* reference.

^a^The power estimate is missing if effect estimates or standard errors could not be retrieved from the publications. If multiple significant effect sizes are reported from different studies, the smallest power estimate is shown. See also Supplementary Table 1 for more details.

^b^GWAS study.

Furthermore, in addition to case/control analyses we also analyzed associations with quantitative traits related to BPD, namely, duration of oxygen supplements, mechanical ventilation, and continuous positive air pressure (CPAP).

## Methods

### Subjects and definition of BPD

In this study, we pooled subjects from two large study groups, namely, PROGRESS (283 preterm infants recruited from 2000 to 2011^19^ and the German Neonatal Network (GNN; 1485 preterm infants recruited from 2008 to 2010).^20^ Ethnic background was available from mothers only. Children from African and Asian mothers were excluded. The remaining subjects were categorized into German, non-German European (including Russian), and Turkish/Middle East genetic background of mothers. Different definitions of BPD were applied in the PROGRESS study and the GNN: The PROGRESS network included preterm infants with GA <32 weeks and excluded those showing severe malformations, administration of oligo- or anhydramnion >3 weeks before birth, or the presence of a severe metabolic disorder. In GNN, preterm infants with GA ≤36 + 6 weeks and a BW <1500 g were included. We harmonized these criteria by including only preterm infants with GA up to 32 weeks. Additionally, we included only preterm infants with high-quality genetic data, resulting in 1061 samples (for genetic filter criteria, see section “Genotyping and imputation”). Furthermore, we consistently applied a definition for mild, moderate, and severe BPD according to the definition of the National Institutes of Health^21^ for preterm infants in both studies. Thereby, preterm infants with supplemental oxygen ≥28 days are defined as BPD with the following degrees: Mild: no need for oxygen supplementation at 36 weeks postmenstrual age (PMA); Moderate: treatment with <30% oxygen after 36 weeks PMA; Severe: ≥30% oxygen and/or positive pressure (mechanical ventilation/CPAP) after 36 weeks PMA. In the case of deaths before 36 weeks PMA, patients with supplemental oxygen ≥28 days were considered moderate when treated with oxygen (<30%) at the time of death and severe when treated with ≥30% oxygen and/or positive pressure at the time of death. Of the 39 deceased patients, 20 deaths occurred before 36 weeks PMA and were considered moderate BPD according to our criteria. We focused on moderate and severe BPD, since heritability was estimated to be smaller for mild BPD.^22^ Hence, in our study, case status was defined by requirement for supplemental oxygen or mechanical ventilation after 36 weeks PMA or until death. For defining controls, we applied the following criteria: survival until 37th week PMA and no supplemental oxygen or mechanical ventilation at 36th week PMA. Using these definitions, we identified 670 controls (including 411 without BPD and 259 mild BPD patients) and 278 cases (including 140 moderate and 138 severe cases) in our studies. The remaining 113 samples of unclear classification were not considered in the analysis of case/control status but were included in quantitative trait analyses.

For the quantification of respiratory support, days of supplied oxygen, days of mechanical ventilation, and days of CPAP were considered, thereby excluding patients deceased before 36 weeks PMA. These measures were assessed as previously described.^19, 20^ In total, information on supplied oxygen, mechanical ventilation, and days of CPAP were available for 989 (93.2%), 1029 (97%), and 982 (92.6%) of the 1061 samples, respectively (see also Table 2 for more details and Supplementary Fig. 1 for a correlation plot). All studies were approved by local Ethics committees, and legal representatives of study subjects provided written informed consent to the study.

**Table 2:** Characterization of the study cohort.

Characteristics	Total (*N* = 1061)	Controls (*N* = 670)/(*N* = 670)	Cases (*N* = 278)	*p* Value
GA, mean weeks (min; max; s.d.)	27 (22.3; 34.9;2.2)	27.2 (22.3; 32; 2.2)	26.3 (22.3; 32; 2)	1.79 × 10^−7^
BW, mean in g (min; max; s.d.)	843 (270; 1700;270.9)	884 (370; 1700; 265)	748.8 (270; 1490; 252.5)	1.15 × 10^−10^
SGA, *n* (%)	290 (27.3)	165 (24.6)	88 (31.7)	0.0278
Sex, *n* (%) female	469 (44.2)	303 (45.2)	116 (41.7)	0.325
Mother country				
Germany, *N* (%)	68 (6.4)	578 (86.3)	242 (87.1)	0.75
Other European, *N* (%)	919 (86.6)	44 (6.6)	17 (6.1)	0.8
Turkey, *N* (%)	746 (7)	48 (7.2)	19 (6.8)	0.859
Twins, *N* (%)	297 (28)	192 (28.7)	76 (27.3)	0.68
Deaths, *N* (%)	39 (3.7)	3 (0.4)	31 (11.2)	4.65 × 10^−8^
Suppl. oxygen, mean days (min; max; s.d.)	53.1 (0; 323; 46.7)	27.8 (0; 86; 25.3)	93.5 (3; 323; 47.7)	<2 × 10^−16^
Mechanical ventilation, mean days (min; max; s.d.)	15.9 (0; 281; 25.0)	9.1 (0; 85; 13.1)	30.6 (0; 281; 38.1)	8.74 × 10^−14^
CPAP, mean days (min; max; s.d.)	49.0 (0; 435; 37.4)	41 (0; 435; 32)	72.6 (1; 281; 42)	<2 × 10^−16^

Shown are phenotype characteristics including duration of respiratory support (namely supplemental oxygen *Suppl. Ox*.), mechanical ventilation and CPAP. Significance levels are calculated considering relatedness. Note that not all individuals with information on respiratory support could be classified in cases or controls.

*GA* gestational age, *BW* birth weight, *SGA* small gestational age, *min* minimum, *max* maximum, *s.d.*standard deviation

### Genotyping and imputation

Genotypes were determined by a custom Affymetrix axiom array. Briefly, this custom array design extends the standard Axiom™ Genome-Wide CEU 1 Array design (ca 580,000 markers) by adding (a) SNPs from the genome-wide association study (GWAS) catalog,^23^ (b) reported expression quantitative trait loci (eQTLs) in the Chicago-eQTL-Browser,^24^ and (c) markers from candidate genes reported for diagnoses such as BPD, respiratory distress syndrome of the neonate, acute respiratory distress syndrome, pneumonia, sepsis, chronic obstructive pulmonary disease, and asthma in OMIM,^25^ PubGene (www.pubgene.com), or Phenopedia.^26^ Markers from genes provided by Affymetrix relevant for ADME (absorption, distribution, metabolism, and elimination), DMET (Drug Metabolism Enzymes and Transporters), and HLA (Human Leukocyte Antigen) genes were also considered. For this array design, dbSNP-Version 130 and genome build hg18 were used. A lift-over to dbSNP-Version 137 genome build hg19 was also available. The extended design finally resulted in 631,728 analyzable genetic markers. Full details of the array content are available upon request from the authors. Intensity files of successfully genotyped samples were combined and genotypes were called by Affymetrix Power Tools version 1.15 and the Axiom GT1 algorithm assuming generic priors. Samples with low call rate of autosomal SNPs (call rate <98%), samples with outlying mean squared difference of the individual’s genotype and population average genotype, sample duplicates, pairs of samples with discordant estimated and reported relatedness, and samples with discordant estimated and reported sex were excluded (excluding 187 individuals, with 1061 remaining). Reported ethnic origin was validated by principal components analysis. For this, a drop-one-in procedure was chosen including 85 European, 88 African, and 97 Asian individuals from the 1000 Genomes project (phase 1, release 3) as reference. For imputation, SNPs were selected according to the following criteria: not monomorphic, call rate ≥97%, *p* value of the deviation from Hardy–Weinberg equilibrium >10^−6^ (strata defined by mother’s country of origin^27^) and *p* value of plate association test >10^−7^. This resulted in 575,367 variants. Imputation was performed using 1000 Genomes phase 1 version 3^28^ as reference (genome built hg-19/ dbSNP 135) and IMPUTE2 v2.3.0^29^ for genotype estimation, after prephasing with SHAPEIT v2.r778.^30^ This resulted in 9,309,859 imputed variants with minor allele frequency (MAF) ≥0.01 and impute info score ≥0.5.

### Retrieval of candidate associations from the literature

To identify appropriate candidate studies, we searched for the term “Bronchopulmonary Dysplasia” in Phenopedia^26^ listed in the online Human Genome Epidemiology encyclopedia. Additionally, PubMed (U.S. National Library of Medicine and the National Institutes of Health) was searched for the MeSH terms “bronchopulmonary dysplasia” and “single nucleotide polymorphisms.” Results were manually screened for candidate gene studies on BPD that considered genes with an immunological role (see Supplementary Table 1 for details). We also screened all studies cited in the identified publications. All nominally significant associations (*p* ≤ 0.05) with BPD or a BPD-related phenotype were considered for replication analysis in our study. We adopted the assignment of SNPs to genes as provided by the reporting study. To improve comprehensiveness, we searched PubMed again, this time using identified candidate genes of the first search round and the MeSH term “bronchopulmonary dysplasia” as search terms. Our search for candidate studies and GWASs was performed in November 2016 and updated in January 2020. Only autosomal associations were considered. This search resulted in 40 candidate studies in which 68 variants within 49 genes were reported (see Supplementary Table 1). After filtering for SNPs that could be unequivocally assigned to a biallelic dbSNP build 135 variant, 61 SNPs within 44 genes remained in analysis. Of these, 55 SNPs within 40 genes were amenable for candidate SNP replication analysis in our cohort, as they were included in the 1000 Genomes imputation reference or found in linkage disequilibrium (LD) with *R*^2^ ≥ 0.5 with a variant fulfilling our post-imputation quality-control criteria (MAF ≥0.01, info score ≥0.5). We consider support for previously reported genetic associations if at least a nominal association with a BPD-related phenotype is found in our data but exclude those with a reported effect direction that does not match the effect direction in our data. Reported genes were classified in six immunological-related subgroups (see Supplementary Table 1): extracellular processes, cellular processes, cytokines, recognition, cell adhesion, and others. The subgroup “others” comprised of genes of various immunologic subtopics not included in the former five categories. The function of the encoded protein served as the basis for this evaluation.

Going beyond direct replication of reported associations, we additionally analyzed all available variants within or near the reported 39 candidate genes. For this purpose, we considered variants fulfilling the following conditions: located within the gene or ±5000 bases 5’ or 3’ of the beginning/start of the gene (positions according to the UCSC genome browser, track UCSC genes,^31^ genome built hg19), MAF ≥0.01 and info score ≥0.5. In total, 8681 SNPs were included in this refined analysis (see Supplementary Tables 2 and 3 for details on SNP and gene-wide analysis).

### Statistical analysis

We calculated a genetic correlation matrix by estimating pairwise relatedness from autosomal SNPs as described.^32^ The resulting matrix was transformed to positive semidefinite by setting negative eigenvalues to the smallest observed positive value. We used this correlation matrix to account for relatedness and ethnicity when comparing phenotypes in Table 2. We applied function “relmatGlmer” of package “lme4qtl”^33^ for this purpose.

Genetic associations were tested by regression modeling. We used gene doses of SNPs, i.e., assumed an additive mode of inheritance. We adjusted for sex, GA, small for gestational age (SGA, defined as BW less than the 10th percentile among infants in the same GA), and mother country. Again, analyses were adjusted for mixed ethnicities in combination with relatedness using a mixed model approach (function “polygenic” of the “GenABEL” package of R^34^ as previously described^35^). Accordingly, binary traits were analyzed as covariate and relatedness adjusted residuals of a linear model.^35^ Furthermore, identified nominal associations were analyzed in a BPD-trend test using the four categories: no BPD, mild, moderate, and severe BPD. This was performed again with the function “relmatLmer” of package “lme4qtl”^33^ with significance calculated with an approximate *F*-test based on the Kenward–Roger approach as implemented in the R-package pbkrtest.^36^ Genetic association results showed no signs of general inflation (maximum lambda was 1.01 observed for the case/control analysis). Gene-based association was carried out by applying locus scoring as implemented in FastBat^37^ using default parameters. Thereby, genotypes of included samples were used to calculate LD, and false discovery rates (FDRs) were calculated using the method of Benjamini and Hochberg.^38^ For regional association plots, LocusZoom^39^ was used. For comparison of observed and expected minimum *p* values (across all four phenotypes, namely, supplied oxygen, mechanical ventilation, CPAP, and case/control state), we compared quantiles of the observed distribution with quantiles of a beta (*α* = 1, β = 4) distribution. For functional annotation of SNPs, we used RegulomeDB^40^ and SNiPA^41^, which also included information on SNP deleteriousness quantified as CADD (Combined Annotation Dependent Depletion) scores.^42^

## Results

### Description of study subjects

Table 2 provides an overview of characteristics of our studied 1061 preterm infants comprising 670 controls and 278 BPD cases. The mean GA was significantly lower, the number of infants small for their GA was significantly higher, and significantly more deaths were observed in cases than in controls. The duration of supplemental oxygen, mechanical ventilation, and CPAP were also significantly higher in cases. When comparing infants with mild, moderate, and severe BPD, we observed an increasing percentage of males (53.7, 55, and 61.6%, respectively).

### Association of previously reported SNPs

We analyzed 55 candidate SNPs within 40 candidate genes for which BPD associations were reported in the literature, 26 of them were directly genotyped while 29 were imputed with high quality as expressed by the imputation info score (see Supplementary Table 2). Our study was sufficiently powered to replicate the effect sizes reported in all original candidate studies and the top hits of reported GWASs. Seven nominally significant associations in seven genes were found in our study. All these hits were well imputed (info score of 1) and frequent, i.e., MAF was >5% (Table 3). Two of these associations (rs2542571-*ERLEC1*and rs2235587-*PSMF1*) showed directions inconsistent when compared with the originally reported association and therefore were not considered as supportive evidence for the originally reported association (Table 4).

**Table 3:** Candidate SNPs with nominally significant associations in quantitative or case/control association analysis in our study.

SNP-count. allele	Gene	Subgroup	CADD	Supplemental oxygen		Mechanical ventilation		CPAP		BPD case/control		BPD-trend test	
				*p* Val.	Effect size (CI95)	*p* Val.	Effect size (CI95)	*p* Val.	Effect size (CI95)	*p* Val.	Effect size (CI95)	*p* Val.	Effect size (CI95)
rs11265269-T	CRP	Recognition	**3.1**	**0.005**	**−0.217 (−0.37 to −0.067)**	**0.025**	**−0.160 (−0.3 to −0.02)**	0.468	−0.035 (−0.13 to 0.059)	0.128	−0.185 (−0.42 to 0.053)	**0.014**	**−0.134 (−0.24 to −0.028)**
rs1427793-T	NUAK1	Cell adhesion	8.7	**0.009**	**−0.201 (−0.35 to −0.05)**	0.972	0.003 (−0.14 to 0.15)	0.132	−0.074 (−0.17 to 0.022)	0.083	−0.212 (−0.45 to 0.028)	**0.013**	**−0.137 (−0.243 to −0.03)**
rs2229569-G	SELL	Cell adhesion	**26**	0.775	−0.026 (−0.21 to 0.15)	**0.027**	**−0.196 (−0.37 to −0.023)**	0.235	0.070 (−0.045 to 0.18)	0.133	0.223 (−0.068 to 0.51)	0.266	0.074 (−0.057 to 0.205)
rs1883617-A	VNN2	Cell adhesion	5.0	0.702	0.027 (−0.11 to 0.17)	0.298	−0.070 (−0.2 to 0.062)	0.122	0.070 (−0.019 to 0.16)	**0.027**	**0.250 (0.029 to 0.47)**	**0.010**	**0.13 (0.031 to 0.229)**
rs2542571-G	ERLEC1	Cellular processes	6.1	0.318	0.069 (−0.066 to 0.2)	0.213	0.080 (−0.046 to 0.21)	0.91	0.005 (−0.08 to 0.09)	**0.033**	**0.230 (0.019 to 0.44)**	**0.036**	**0.102 (0.007 to 0.196)**
rs2235587-A	PSMF1	Recognition	0.0	0.571	−0.055 (−0.24 to 0.13)	**0.035**	**−0.191 (−0.37 to −0.014)**	0.218	−0.076 (−0.2 to 0.044)	0.785	−0.041 (−0.34 to 0.25)	0.704	−0.026 (−0.158 to 0.107)
rs4148913-A	CHST3	Cell adhesion	**13.1**	0.208	0.085 (−0.047 to 0.22)	**0.037**	**0.131 (0.0079 to 0.25)**	0.795	−0.011 (−0.09 to 0.07)	0.575	−0.060 (−0.27 to 0.15)	0.575	−0.027 (−0.12 to 0.067)

This table shows the results of the analysis of previously reported SNPs from Table 1 with at least nominal significance in our association analyses. Results in bold highlight significant associations. BPD-trend tests for an increasing or decreasing trend in allele frequency across the four groups no, mild, moderate, and severe BPD. Allele: minor/major allele.

*CPAP* continuous positive air pressure, *CI95* 95% confidence interval.

**Table 4:** Comparison of the reported and observed effect sizes of nominally replicated candidate SNP associations.

SNP	Gene	Subgroups	Alleles Lit.	Effect size Lit. (OR)	Phenotype Lit.	Allele study	Effect size study (beta)	Phenotype study	Effect direction consistency Lit. vs. study
rs11265269	CRP	Recognition	C (T)	1.82	C/C	T (C))	−0.217	Suppl. Ox.	Consistent
							−0.160	Mech. Vent.	Consistent
							−0.134	BPD-trend test	Consistent
rs1427793	NUAK1	Cell adhesion	NA	NA	C/C	T (C)	−0.201	Suppl. Ox.	NA
							−0.137	BPD-trend test	NA
rs2229569	SELL	Cell adhesion	A (G)	2.45	C/C	G (A)	−0.196	Mech. Vent.	Consistent
rs1883617	VNN2	Cell adhesion	A (G)	1.21	C/C	A (G)	0.250	Case/control	Consistent
							0.13	BPD-trend test	Consistent
rs2542571	ERLEC1	Cellular processes	G (T)	0.8	C/C	G (T)	0.230	Case/control	Inconsistent
							0.102	BPD-trend test	Inconsistent
rs2235587	PSMF1	Recognition	A (G)	1.38	C/C	A (G)	−0.191	Mech. Vent.	Inconsistent
rs4148913	CHST3	Cell adhesion	G (A)	0.79	C/C	A (G)	0.131	Mech. Vent.	Consistent

The reference allele is shown in parentheses. Inconsistent directions of effects were observed for the SNPs in PSMF1 and ERLEC. For NUAK1, no effect size information was reported, i.e., no comparison was possible. BPD-trend tests for an increasing or decreasing trend in allele frequency across the four groups no, mild, moderate, and severe BPD.

*Lit.* literature, *NA* effect direction not available in the original study, *OR* odds ratio, *Mech. Vent*. mechanical ventilation, *Suppl. Ox*. supplemental oxygen.

The strongest association was found for the SNP rs11265269-*CRP* with supplemental oxygen (*p* = 0.005). Additional associations were found with mechanical ventilation (*p* = 0.025) and by the BPD-trend test (*p* = 0.014). C-reactive protein (CRP) is a well-known inflammatory marker and plays a major role in the body’s immune response.^43^ Effect direction of associations was consistent with the literature,^12^ with major allele T showing a protective effect. This variant is located 44 kb upstream of *CRP* and is according to SNiPA strongly associated with CRP levels in the UK Biobank (*p* = 5.35 × 10^−14^).

The second strongest finding was the SNP rs1427793 in *NUAK1* (NUAK Family Kinase 1) associated with supplemental oxygen (*p* = 9.18 × 10^−3^). Among others, NUAK1 is involved in cell adhesion, i.e., important for relocation and movement of blood cells, including leukocytes, macrophages, and others.^44^ The corresponding case/control *p* value closely missed significance (*p* = 0.08). The consistency of effect directions with the literature could not be checked due to missing information in the original study. According to SNiPA, rs1327793-*NUAK1* is a 3′-untranslated region variant. It was observed to affect transcription factor-binding (site ID ENSR00000436389) and histone methylation in several tissues, including the lung and blood. RegulomeDB classified this variant as class 4, i.e., weak evidence regarding binding of regulatory elements. The CADD score for this SNP was 8.7, i.e., moderately large. For this association, a stronger effect was observed in females (*p*_interaction_ = 0.006, Supplementary Fig. 2), while all other candidate variants did not show significant different effects between sexes.

Another nominally associated SNP with consistent effect direction was rs2229569-*SELL* (L-Selectin), a coding missense variant (proline > serine, *p* = 0.027 with mechanical ventilation). SELL is a cell surface protein involved in cell adhesion.^45^ Major Allele G was associated with less requirement of mechanical ventilation and, accordingly, shows a protective effect regarding BPD. SNiPA reported effects on transcription (cis-eQTL on *SELL* observed in blood, and also on *SCYL3*, a gene also relevant for cell adhesion). RegulomeDB evaluated the variant as class 4. Expression of SELL was reported for a variety of tissues, e.g., lung tissue and lymphocytes. High evidence for a functional consequence is indicated by its CADD score of 26.

Furthermore, variant rs1883617-*VNN2* showed significant consistent association with BPD in the BPD-trend test and in case/control analysis (*p* = 0.027, and 0.01, respectively). When restricting controls to patients without BPD, nominal association remained (*p* ≤ 0.05). VNN2 is involved in transendothelial migration of neutrophils.^46^ The encoded gene product is a GPI-anchored cell surface molecule that takes part in transendothelial migration of neutrophils. According to SNiPA, the genetic variant is a missense coding variant, affecting amino acid sequence of *VNN2*. The corresponding CADD score of 16.55 is high, suggesting a functional relevance of the variant. RegulomeDB ranked it high with “1d”, i.e., evidence for regulatory effects from eQTL, transcription factor binding, motif, and DNase peak data.

Finally, we observed rs4148913-*CHST3* (*p* = 0.037) to be nominally associated with the duration of mechanical ventilation with consistent effect direction. *CHST3* is involved in cell adhesion by synthesizing chondroitin 6-sulfate^47^ and supposed to play a role in the maintenance of naive T lymphocytes in the spleen.^48^ Results on SNiPA implicated a direct regulative effect for rs4148913-*CHST3* on *SPOCK2*. RegulomeDB reported class 4 again and the CADD score was 13.1, suggesting functional relevance of the variant.

Interestingly, 4 in these 5 genes (80%) are at least partly involved in processes related to “cell adhesion” (*SELL*, *NUAK1*, *VNN2*, and *CHST3*). This is more than expected by chance, as only13% of all analyzed genes (Supplementary Table 1) were classified as cell adhesion genes (*p* = 0.004, Fisher’s exact test).

### Association analyses of additional variants of previously reported genes

**Fig. 1:**
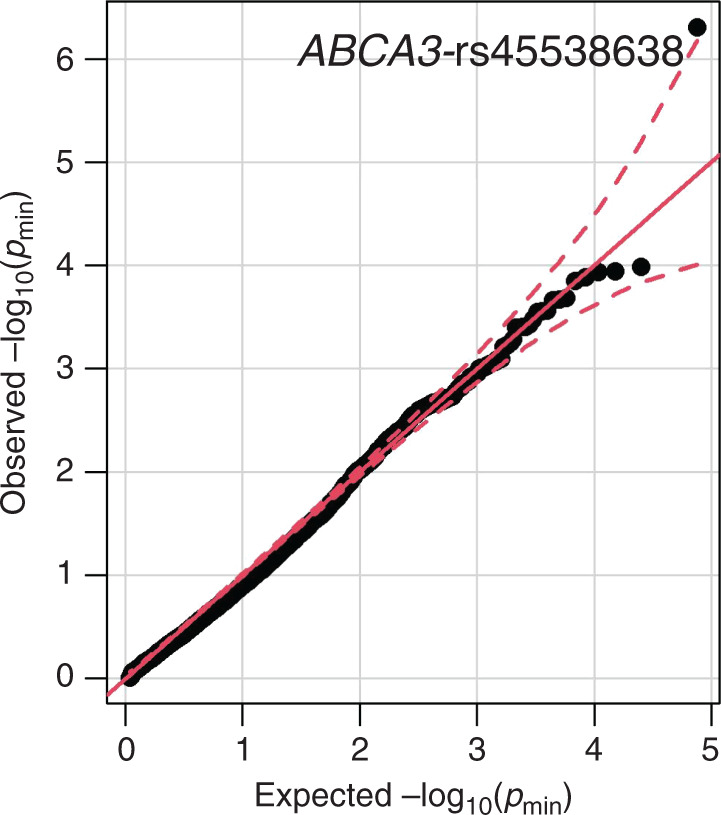
Quantile–quantile plot comparing observed and expected *p* value minimum of SNP associations with the four investigated phenotypes supplied oxygen, mechanical ventilation, CPAP, and case/control state. A total of 9469 SNPs in or near 49 candidate genes were investigated. One variant rs45538638-ABCA3 was stronger associated with CPAP than expected by chance accounting for the tested number of SNPs and phenotypes.

Going beyond direct replication of reported associations, we additionally analyzed all available variants within ±5000 bases 5’ or 3’ of the reported 49 candidate genes, including 9 more genes in which the variants reported in the literature could not be mapped to our genetic data. From the investigated 9469 SNPs, we observed one association withstanding multiple testing correction (see Table 5 and Fig. 1), rs45538638-*ABCA3* (ATP-binding cassette sub-family A member 3). This variant was associated with CPAP (*p* = 4.9 × 10^−7^) corresponding to a (multiple testing corrected) FDR of 0.0046. The SNP is also associated with the need for supplemental oxygen (*p* = 0.0036), case/control status (*p* = 0.0078), and the BPD-trend test (*p* = 0.01), and shows trend association with mechanical ventilation (*p* = 0.06). The minor allele T is protective, i.e., associated with a decreased need for CPAP and supplemental oxygen and a decreased risk for BPD. No relevant LD with the variant rs13332514 originally reported for *ABCA3* was observed because this variant is located close to a recombination hotspot of the haploblock containing rs45538638-*ABCA3* (see Supplementary Fig. 3). No other SNP in LD with rs45538638-*ABCA3* was included in our study, which is reasonable as rs45538638 was one of the additionally included SNPs in the framework of the custom design of our SNP array. Extending LD analysis to all variants included in 1000 Genomes phase 3 version 5 within 1 Mb, no variant in relevant LD was found either (Supplementary Table 4). RegulomeDB classified rs45538638-*ABCA3* as class 2b, i.e., likely to affect binding of transcriptional factors N-Myc, Myc, and CLOCK:BMAL whose binding sites were altered by the SNP. Moreover, allele-specific chromatin modifications were predicted. Phenotypes between carriers and non-carriers of rs45538638-*ABCA3-T* did not differ significantly in other clinical traits like GA or SGA (Supplementary Table 5).

**Table 5:** Associations of rs45538638-ABCA3 with different BPD-related phenotypes.

Phenotype	Effect size (CI95)	*p* Value
Supplemental oxygen	−0.67 (−1.1 to −0.22)	0.004
Mechanical ventilation	−0.42 (−0.85 to 0.016)	0.06
CPAP	−0.74 (−1.0 to −0.45)	4.9 × 10^−7^
Case/control	−0.97 (−1.7 to −0.26)	0.008
Trend test	−0.414 (−0.73 to −0.094)	0.011

Association of this variant with CPAP was significant also after adjusting for multiple testing (FDR = 0.004).

*CI95* 95% confidence interval.

Gene-based locus scoring did not identify additional associations when correcting for multiple testing (Supplementary Table 6). To ensure robustness of our findings, we repeated association analyses excluding all 20 patients who died before 36 weeks PMA. In this sensitivity analysis, effect sizes were very similar to those of our main analysis and all identified associations remained statistically significant (Supplementary Table 7).

## Discussion

The aim of this study was to perform a comprehensive replication analysis of reported genetic associations of BPD focusing on immunologically relevant candidate genes in a relatively large cohort of 1061 patients. We not only aimed to replicate the originally reported SNPs but also analyzed the reported genes in detail by considering other common SNPs of these genes. We were able to replicate five variant associations with effect directions not conflicting the original studies and found a new association at *ABCA3*.

We focus on candidate analyses rather than hypothesis-free approaches to have a reasonable power. The power of our analysis was typically >90% to replicate associations reported in previous candidate studies and the top hits of reported GWAS studies. It was also sufficient to have a decent chance of replicating suggestive candidates of GWAS studies (Table 1).

Going beyond case/control analysis, we also analyzed quantitative measures related to BPD because quantitative analyses are typically better powered. Difficulties in replicating BPD associations were also reported previously.^8^ Besides the above-mentioned power issue of binary traits, other explanations for this lack of direct replication are conceivable, namely, small sample sizes of reporting studies ranging from 46 to 1726, heterogeneity in the definition of case/control status across studies, publication bias of reported associations, and complexity of the phenotype, i.e., the possibility that there are no larger but many smaller genetic effects that cannot be detected with the currently available sample sizes as observed for other traits.

Of note, three of the variants associated with effect directions not conflicting the original studies were previously reported in a GWAS analysis (rs11265269-*CRP*,^12^ rs1427793-*NUAK1*,^49^ and rs4148913-*CHST3*^8^) but none achieved genome-wide significance. Hence, their finding required further validation, which is achieved by our study to some extent.

However, it needs to be acknowledged that only nominal significance in replication of candidate SNPs was achieved. More robust confirmation is desirable as well as clarifying the role of the genes in the pathophysiology of BPD development.

When extending our analysis to all genotyped or imputed SNPs located in or close to the 39 previously reported candidate genes, SNP rs45538638-*ABCA3* showed strong association with CPAP length withstanding stringent correction for multiple testing (*p* = 4.9 × 10^−7^, FDR = 0.004, Fig. 1). *ABCA3* is a membrane-associated ATP-binding transporter of the ABC A-subfamily,^50^ predominantly expressed on intracellular lamellar bodies of lung alveolar type II cells of the developing and mature lung^51, 52^ where it is supposed to be involved in maturation of alveolar type II cells, synthesizing surfactant^53^ and interstitial lung fibrosis.^54^ A link of this gene to respiratory distress syndrome (RDS), a preliminary stage of BPD,^55^ was also described.^56^ The molecular mechanism of this association remains to be clarified. There are several transcription factors serving as candidates: binding sites of N-Myc, Myc, and CLOCK:BMAL are possibly affected by the associated variant according to RegulomeDB. Furthermore, transcription factors binding upstream can also be considered as candidates, including *TTF-1* increasing gene expression of *ABCA3* with relevance for lung development.^57, 58^ Although another study could not find any significant associations of TTF-1 variants with BPD,^59^ a possible connection and thus an importance of this transcription factor for the development of BPD remains. Still, the evidence of this finding does not yet allow final conclusions: on the one hand, data quality of rs45538638 was high, as it was directly genotyped on the chip. On the other hand, the MAF for allele T is only 0.022, which might cause spurious associations. Of note, in the original study which reported an association of *ABCA3* with BPD,^52^ variant rs45538638-*ABCA3* was not analyzed since these authors excluded all variants with MAF <0.1. Instead, they found an association of rs13332514-*ABCA3* with requirement of supplemental oxygen at 28 postnatal days in very preterm infants (odds ratio 4.2 95% confidence interval (1.7–10.8), *p* = 0.002). No association was seen for this SNP in our study and rs13332514-*ABCA3* showed no relevant LD with rs45538638-*ABCA3* (see Supplementary Fig. 2).

One limitation of our study is that our findings cannot be readily applied to distant ethnicities, as our study design focused on probands of Caucasian descent. A second limitation is that associations with BPD may have been missed because the analysis was limited to certain candidate genes. However, given the experiences of previous GWAS for BPD and our sample size, we consider a candidate study as more appropriate to reduce the burden of multiple testing. Moreover, phenotype definitions differed between studies and standard errors are not always provided. Therefore, we chose replication analysis as our main study design. Larger studies and—as more genetic studies emerge—meta-analyses are required for a comprehensive hypothesis-free search for genetic associations of this rare phenotype, though alternative study designs such as gene-based analyses of genetically imputed expression data^60^ and pathway-based analyses are conceivable. In studies with sufficient sample size, a split design could also be considered. A further limitation is the adoption of SNPs to gene assignment reported in the original studies. These assignments are typically based on proximity while functional considerations would be more appropriate. Hence, we cannot exclude that observed associations might relate to other genes than the reported ones. Another limitation is that we only considered common variants. Consideration of rare variants, for example, in the framework of a gene-based analysis could add further associations.^61^ Finally, the definition of BPD was subject to changes over the past decades. In our study, a compromise was chosen to combine the data sets of two large study groups. A future improvement could be to implement an oxygen-challenge test to define BPD as proposed by Walsh et al.^62^

In summary, our results showed only limited support for previously reported candidate genes and SNP associations with BPD. Supporting evidence was found for previously reported SNP associations in or near *CRP*, *NUAK1*, *SELL*, *VNN2*, and *CHST3*. We also found a novel association in the ABC-transporter gene *ABCA3*. Further replications, GWASs of larger sample sizes, and meta-analyses are required to unravel genetics of BPD.

## Supplementary information

Supplementary material

Supplementary material
