# Supplementary material for: Verification of immunology-related genetic associations in BPD supports ABCA3 and five other genes
Source: Pediatr Res. 2021 Aug 31;92(1):190–8. doi: 10.1038/s41390-021-01689-y (PMC9411063; doi:10.1038/s41390-021-01689-y)
Supplement: Supplementary file 1 — Supplementary material [file 41390_2021_1689_MOESM1_ESM.pdf]

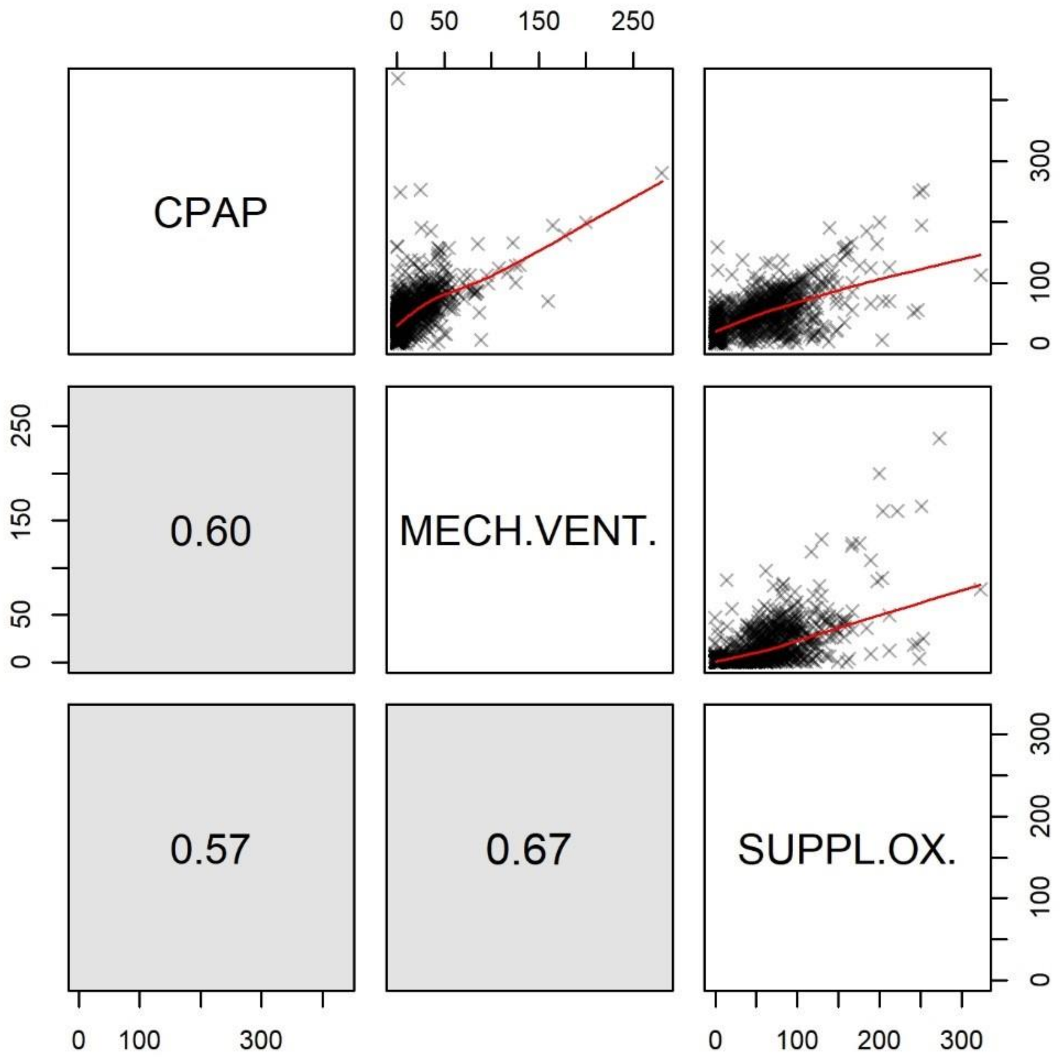

Supplemental figure 1: Correlation between considered quantitative measures of respiratory support

This figure shows the correlation between each quantitative measures of respiratory support (supplemental oxygen, mechanical ventilation and CPAP). All measures are shown in days. The lower triangle shows the value of the Spearman rank correlation rho.

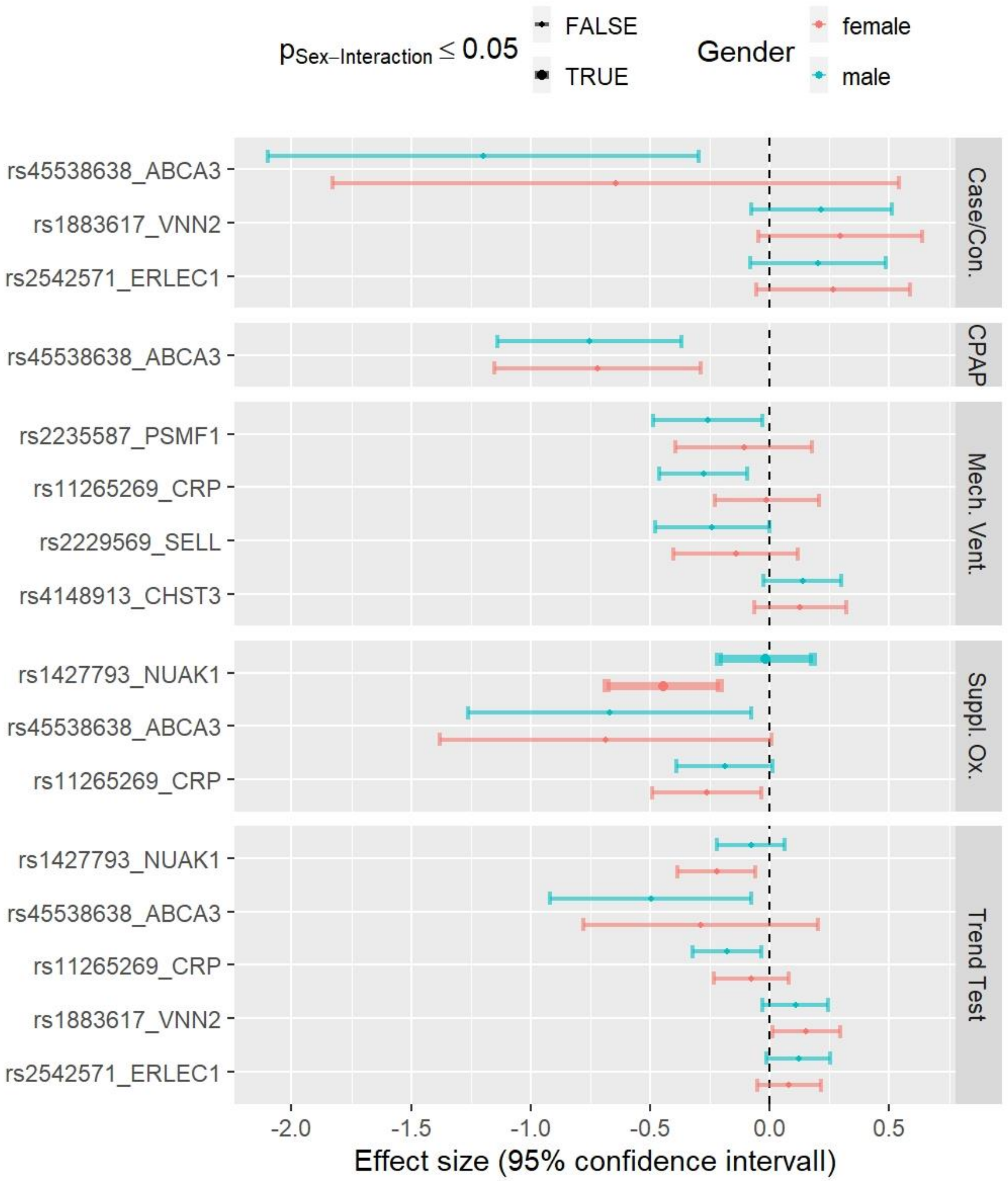

Supplemental figure 2: Sex specificity of observed genetic associations

We compared male-specific and female-specific effect sizes in an interactions test. We observed nominal significance for different effect sizes for the association of rs1427793-NUAK1 with the time of requirement of oxygen, where the effect was stronger pronounced in females (p interaction = 0.006).

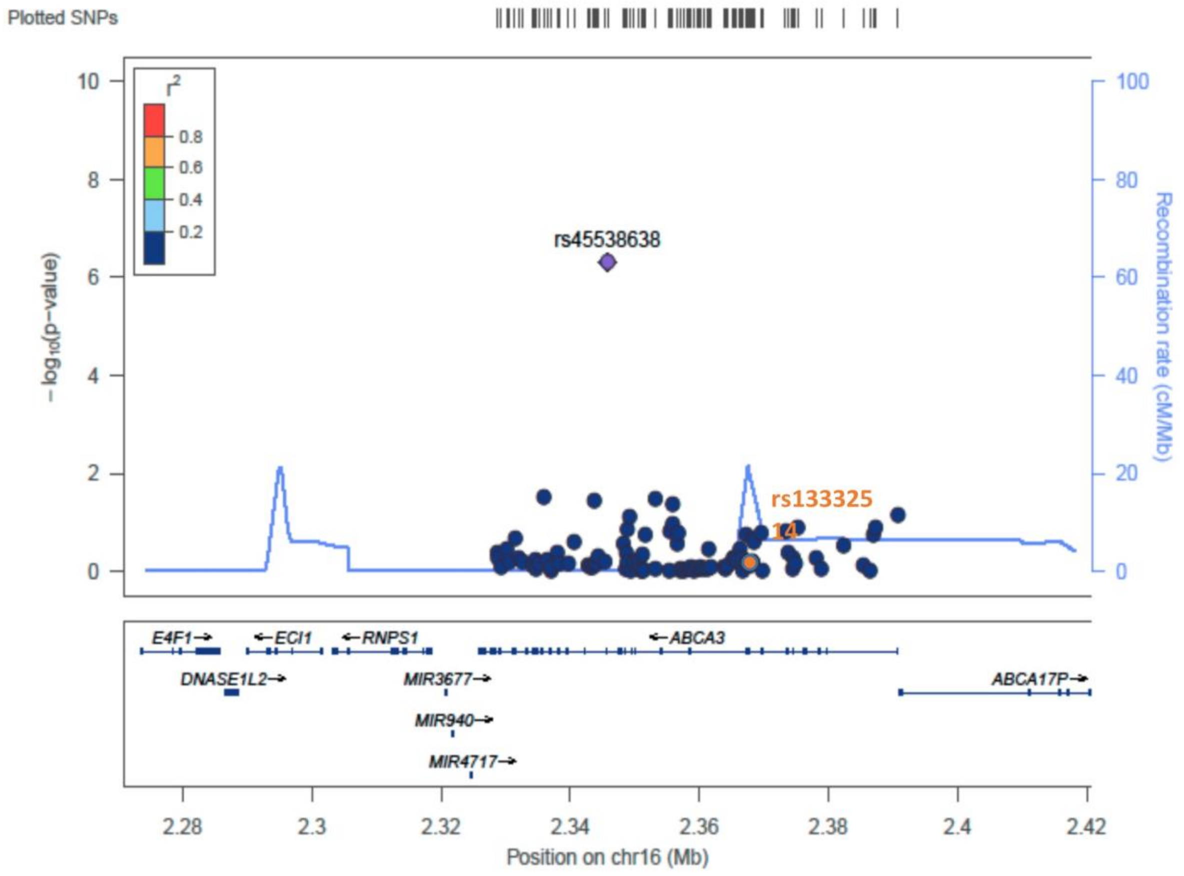

Supplemental figure 3: Regional plot for the association of ABCA3 with CPAP

No variant in LD with rs45538638 was available in the imputation reference panel. Variant rs133325 was previously reported for association with BPD.
